# Novel links between ciliopathies and FGF-related craniofacial syndromes

**DOI:** 10.1186/2046-2530-1-S1-O25

**Published:** 2012-11-16

**Authors:** K Liu, JT Tabler, HL Szabo-Rogers, A Mesbahi, C Healy, W Barrell, B Wlodarczyk, JB Wallingford, R Finnell

**Affiliations:** 1 King's College London, UK; 2 University of Texas Southwestern, USA; 3 University of Texas at Austin, USA

## 

Recent studies suggest that planar cell polarity (PCP) genes coordinate cell polarity, ciliogenesis and signalling during mammalian development. *FUZ* is a PCP gene implicated in human congenital anomalies, including neural tube defects and orofacial clefting. Our analysis of *fuzzy* mutant mice reveals ciliogenesis defects in craniofacial tissues as well as a suite of phenotypes reminiscent of FGF-related craniofacial disorders. Mutants have coronal synostosis, shortened facial extensions, low-set ears and a high-arched palate. To our surprise, we found that the facial defects are due to increased neural crest migration into the first branchial arch (BA1), resulting in maxillary hyperplasia. Furthermore, the neural crest cells migrate in a disorganized fashion, deeper than normal and with fewer cell-cell contacts. This ectopic migration correlates with a dramatic increase in FGF signaling, first in the mid-hindbrain boundary, and then in the BA1 epithelia. The increased tissue causes a medial positional shift in the palatal primordia that manifests as a high-arched palate with pseudo-cleft. Genetic loss of *fgf8* rescues the maxillary hyperplasia. Taken together, our data suggest a novel interplay between ciliogenesis, FGF signalling and migration of neural crest which may underlie congenital craniofacial dysmorphologies.

